# Significant production of humic fluorescent dissolved organic matter in the continental shelf waters of the northwestern Pacific Ocean

**DOI:** 10.1038/s41598-018-23299-1

**Published:** 2018-03-20

**Authors:** Jeonghyun Kim, Hyung-Mi Cho, Guebuem Kim

**Affiliations:** School of Earth and Environmental Sciences, Seoul National University, 1 Gwanak-ro, Gwanak-gu, Seoul, 08826 Korea

## Abstract

Fluorescent dissolved organic matter (FDOM) is important for marine organisms and the global carbon cycle contributing to the optical properties of surface seawater and organic carbon budgets. Rivers are known to be the main source of FDOM in coastal oceans and marginal seas. In this study, however, we show that the contribution of FDOM produced from organic sediments of the northwestern Pacific continental shelf is similar to that from the Changjiang River. FDOM showed relatively higher concentrations at stations off the Changjiang River mouth and in the central Yellow Sea. Based on temperature-salinity diagrams, the major source of surface FDOM in summer surface waters was found to be from the Changjiang River while that observed in the winter water column was produced mainly in the continental shelf. A good correlation between ^228^Ra and the humic-like FDOM (FDOM_H_) during the winter suggests that the FDOM_H_ is produced mainly from marine sediments and enriched in water over the water residence times. A simple mass balance calculation shows that the excess FDOM_H_ fluxes produced from marine sediments account for 30–40% of the riverine source. This result suggests that the continental shelf is an important hidden source of FDOM in the upper ocean.

## Introduction

Colored dissolved organic matter (CDOM) is the light-absorbing fraction of reduced organic matter in aquatic environments. A part of CDOM, which is also fluorescent over a wide wavelength range after absorbing energy, is traditionally termed as fluorescent dissolved organic matter (FDOM). Thus, FDOM has a direct influence on underwater light fields, especially absorbing ultraviolet and visible radiation^1–4^. These also impact the marine chemical cycles in terms of the production of greenhouse gases (e.g., carbonyl sulfide, CO, and CO_2_), speciation of trace metals, and bioavailability of organic substrates through a variety of photochemical reactions^4–6^. FDOM has been used as an indicator of dissolved organic matter (DOM) and dissolved organic carbon (DOC) in the ocean because most DOM show distinct optical signals.

In coastal oceans and marginal seas, rivers are known to be the major source of FDOM as significant negative relationships between FDOM and salinity and lignin have been observed^7,8^. However, recent studies have suggested that sinking organic matter and bottom sediments could also be a significant source of FDOM in the ocean^9–12^. Yamashita and Tanoue^13^ showed that *in situ* production of FDOM in the interior of the Pacific Ocean is almost five times greater than global riverine inputs of terrestrial FDOM. As such, Kim and Kim^14^ showed that the deep-sea production of humic-like FDOM accounts for 20–30% of the riverine flux in the surface ocean of the East Sea (Sea of Japan). More recently, Kim and Kim^9^ showed significant excess humic-like FDOM inputs from bottom sediments based on apparent oxygen utilization (AOU) and FDOM correlations. They showed that approximately 10% of the humic-like FDOM in the deep ocean (1000 m – bottom) can be introduced from the anoxic layer of bottom sediments in the East Sea^9^.

**Figure 1:**
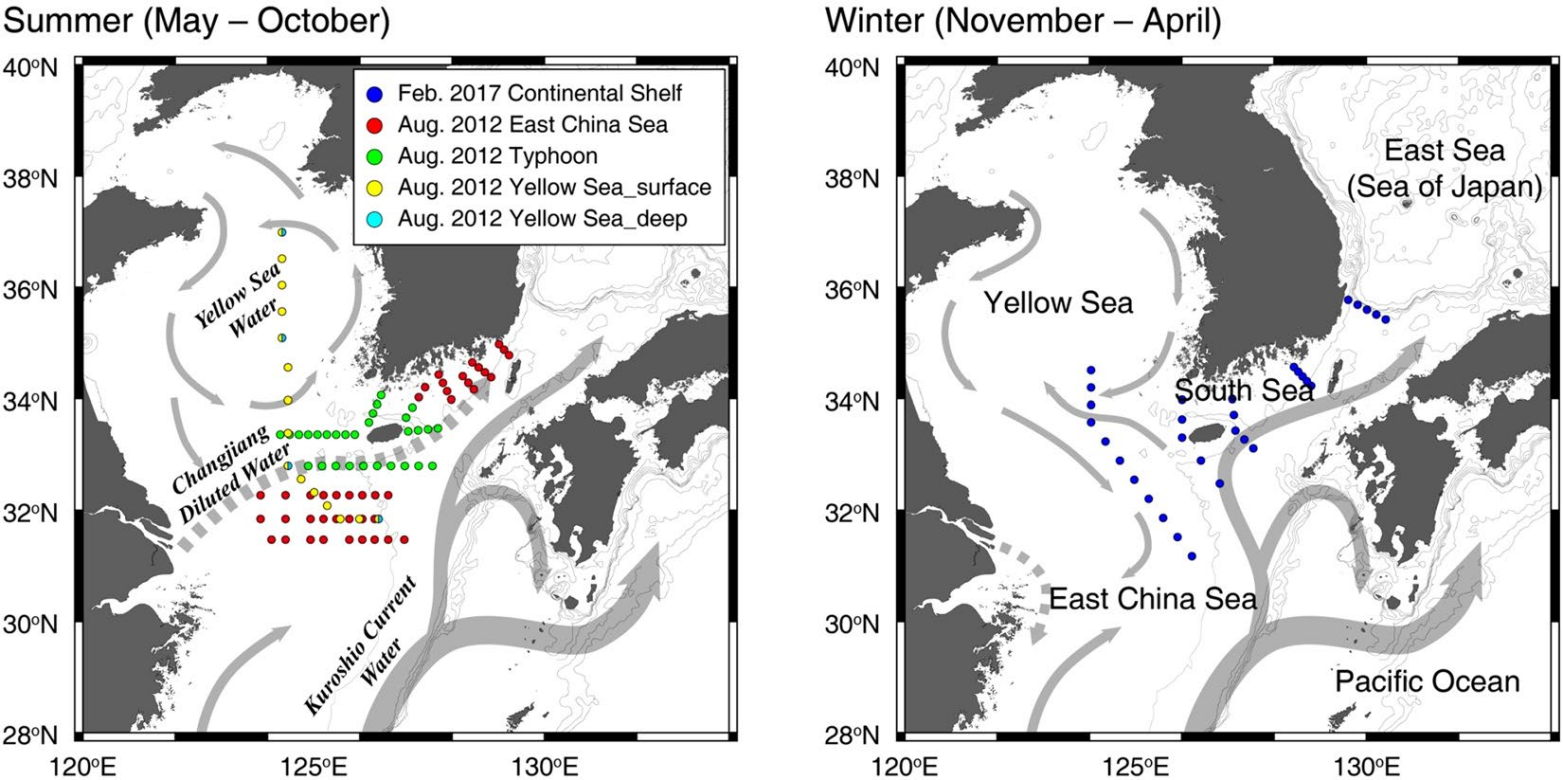
Maps showing sampling stations, bottom topography, and schematics patterns of surface currents on the northwestern Pacific continental shelf during the summer and winter sampling periods. The solid arrows represent the seawater current, and the dotted arrows represent the Changjiang Diluted Water (CDW) current. The maps were created using Ocean Data View software version 4.7.10. (https://odv.awi.de) and the sampling stations and current patterns were drawn using Adobe Illustrator CC software version 2015.0.1. (http://www.adobe.com/).

The northwestern Pacific continental shelf (approximately 1 million km^2^ area), including the Yellow Sea, the East China Sea, the southern sea off Korea, and the East Sea, is one of the largest continental shelves in the world^15^ (Fig. 1). The Kuroshio branch water, which is a strong western boundary current, flows into the northwestern Pacific continental shelf and the East Sea^16^. Here, the branch of the Kuroshio Current is mixed with the river waters and the continental shelf water, thereby supplying high concentrations of terrestrial organic matter and inorganic nutrients. During the warm season (from May to November), the vertical temperature gradient and the freshwater discharge induce a strong pycnocline (surface layer of 10 m)^17^, while it is fully mixed during the cold season (from December to April)^17^. In particular, this continental shelf contains high concentrations of sedimentary organic matter with fine-grained sediments from the Changjiang (Yangtze) and Yellow rivers. The Changjiang River, which is one of the world’s largest rivers, is the dominant source of freshwater (~9 × 10^2^ km^3^/year; approximately 90% of total river discharge) in this region^16,18,19^. The freshwater plume originating from the Changjiang River extends southward along the Zhejiang coast during the winter and northeastward to the East Sea through the Korea-Tsushima Strait during the summer^16,18,20^. After the construction of the Three Gorges Dam in 2003, the sediment load decreased by approximately 30%^21^, while no significant long-term variations in the annual freshwater discharge were observed^22–24^.

The distribution characteristics of FDOM in this region were recently investigated by analyzing the absorption and fluorescence of DOM. Guo *et al*.^25^ reported the conservative and non-conservative distribution behaviors of CDOM versus salinity in Chinese estuaries. Bai *et al*.^26^ and Su *et al*.^27^ investigated the factors contributing to the spatial and seasonal distributions of FDOM in the southern Yellow Sea and the East China Sea using the fluorescence index and the relationships between FDOM components and certain parameters (Chl-a, AOU, and salinity) indicating potential sources.

In this study, we examined the relative contributions of FDOM production from organic sediments compared with those from riverine inputs based on the hypothesis that organic-rich continental shelf sediments can effectively produce FDOM for the shallow water column. We used specific hydrological properties based on the temperature-salinity (T-S) diagram and ^228^Ra as tracers to differentiate different sources of FDOM in the region.

## Results and Discussion

### Identification of FDOM components

Three FDOM components were characterized using the Parallel Factor Analysis (PARAFAC) model (Figure S1). Briefly, Component 1 (C1, Ex/Em = 325/392 nm) is traditionally referred to as the marine humic-like FDOM component (FDOM_H_), M peak^28^. It is known to be derived mainly from microbial-derived fulvic acids^28^. Murphy *et al*.^29^ also reported that this component is *in-situ* produced from terrestrial and marine organic substrates and is a newly-produced organic matter regardless of its origin. The C1 can be produced by the shift of the peak position to shorter wavelengths (“blue-shift”) from C peak showing longer wavelengths. Component 2 (C2, Ex/Em = 285/332 nm) is identified as the tryptophan amino acid-like and protein-like FDOM component (FDOM_P_), T peak^28^. It is known to originate from polyphenolic- or protein-like materials derived from freshly produced organic matter^30^. Thus, the relatively high concentrations of FDOM_P_ were observed in regions where biological activity is relatively high in the surface ocean^31,32^. The FDOM_P_ can also originate from anthropogenic sewage and farm wastes^32^. Component 3 (C3, Ex/Em = 385/448 nm) is the terrestrial FDOM_H_ component, C peak, which has longer excitation and emission wavelengths compared to those of C1^28^. Its red-shifted peak location is known to be associated with its condensed molecular structures, indicating that the fluorescent signal of C3 is linked to terrestrial detrital DOM produced by degradation of higher plants^33^. In addition, the production of C3 from marine organic matter is observed in the global deep ocean^34^. Thus, in this study, we do not differentiate C1 and C3 explicitly, and simply regard them as different components of FDOM_H_.

### The origins of FDOM in the ocean

**Figure 2:**
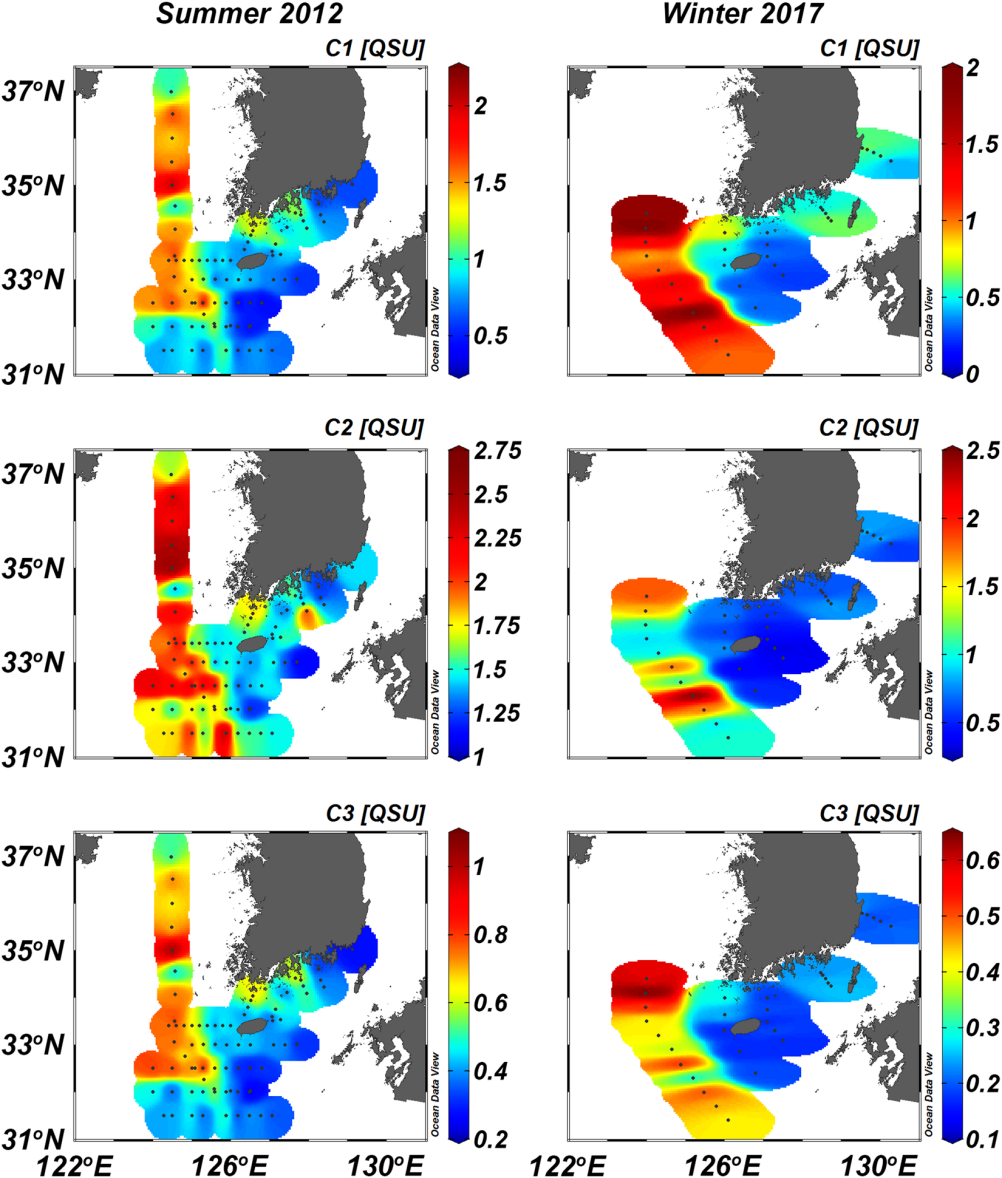
Contour figures of FDOM components, C1, C2, and C3, in surface waters of the northwestern Pacific continental shelf during the summer of 2012 and winter of 2017. The contour plots were created using Ocean Data View software version 4.7.10. (https://odv.awi.de).

**Figure 3:**
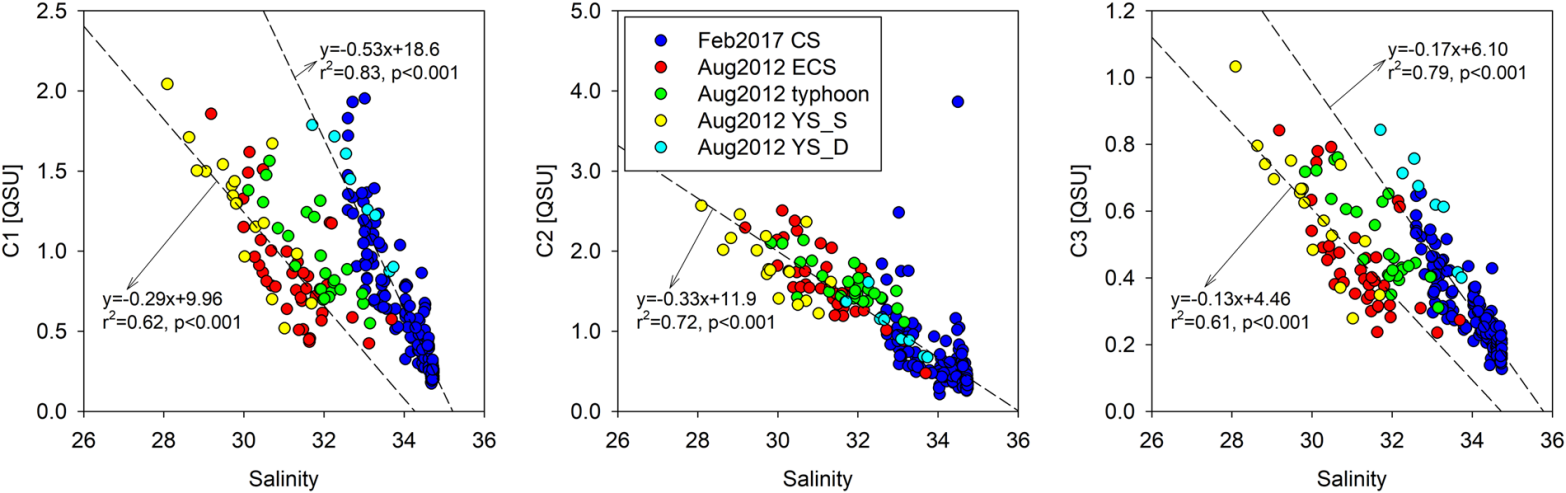
Scatterplots of FDOM concentrations versus salinities in the continental shelf waters during the summer of 2012 and winter of 2017. Correlation coefficients between FDOM_H_ and salinity were evaluated from the data for the surface FDOM during August 2012, except for the post-typhoon period, and for the winter of 2017 and from the deep FDOM of the Yellow Sea during August 2012.

The concentrations of the three FDOM components in surface water were relatively higher at stations off the Changjiang River mouth and in the central Yellow Sea during the summer (Fig. 2). The average concentrations of FDOM in the surface water of the continental shelf (1.0 ± 0.4 QSU for C1; 1.7 ± 0.3 QSU for C2; and 0.5 ± 0.2 QSU for C3) were approximately two times higher than those at the highest salinity station (0.5 QSU for C1; 1.1 QSU for C2; and 0.3 QSU for C3) in the East China Sea, which is considered as a branch of the oligotrophic Kuroshio Current water (Figs 2 and 3). FDOM concentrations were negatively correlated to salinities (Fig. 3). The distribution of FDOM and its relationship to salinity during the summer indicate that FDOM, regardless of its properties, originates mainly from the Changjiang River^14^. Based on the potential T-S diagram for the summer, the distributions of all FDOM components in the surface continental shelf waters showed mixing patterns between the Changjiang Diluted Water (CDW) and the oligotrophic Kuroshio Surface Water (KSW) (Figure S2). This pattern is related to the Changjiang River plume extending northeastward to the East Sea over the continental shelf region during the summer. Changjiang River water contains high concentrations of inorganic nutrients as well as terrestrial organic matter^35^. Efflux of enriched fulvic and humic acids from soils and *in situ* biological degradation of organic matter in the river water may contribute to high concentrations of riverine FDOM_H_^26,33,36^. In addition, river-driven organic and inorganic nutrients could result in active biological production of FDOM_P_ at the river mouth.

During February 2017, the relationship between FDOM and salinity also showed negative trends (Fig. 3). However, these distribution patterns seem to be independent of the direct Changjiang River discharge as the influence of CDW on the study region is minor during the winter (Figure S2)^37^. This hypothesis is supported by the sectional distributions of the FDOM components across the continental shelf from the Yellow Sea to the southern East China Sea during February 2017, showing an increase in FDOM toward the central Yellow Sea (Figures S2; S3). Furthermore, the winter T-S diagram shows that the FDOM distributions are dependent on the mixing between the Yellow Sea Bottom Water (YSBW) and the Kuroshio Tropical Water (KTW). The concentrations of FDOM_H_ during February 2017 were much higher than those expected from the FDOM_H_-salinity correlation observed during August 2012 (Fig. 3). The excess FDOM_H_, which is in excess of that contributed by the Changjiang River, is consistent with a significant enrichment of FDOM_H_ in the deep-water layer of the Yellow Sea observed during August 2012 for the same salinity waters (Fig. 3).

There are three possible processes that influence the enrichment of high FDOM_H_ in the YSBW: 1) inputs from other smaller rivers, 2) submarine fresh groundwater discharge, and 3) *in situ* microbial production or diffusive and advective fluxes from bottom sediments and pore waters. Smaller river and fresh groundwater inputs can be excluded from the major source of deep FDOM_H_ because the Changjiang River is the dominant freshwater source (more than 90%). Even if they are significant, these freshwater-origin FDOM_H_ samples should be shown in the T-S diagram. However, we cannot differentiate internal sources from the sinking particles and bottom organic sediments, which can gradually enhance FDOM_H_ in deep water over time. We do not use AOU as an indicator for *in situ* production as it is not conservative in these seasonally mixed shelf waters.

**Figure 4:**
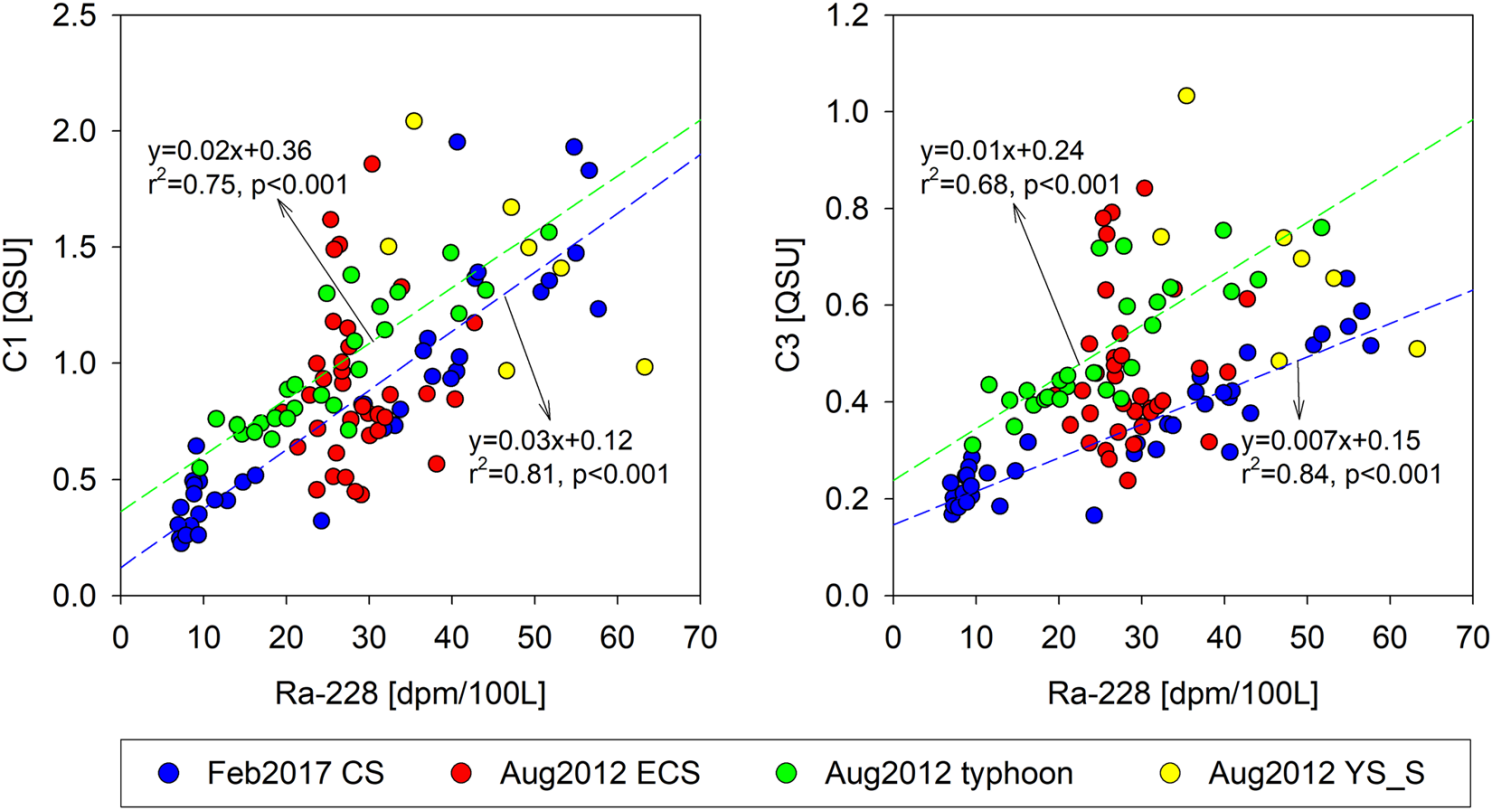
Scatterplots of C1 and C3 concentrations versus the activities of ^228^Ra in the continental shelf waters during the summer of 2012 and winter of 2017.

**Figure 5:**
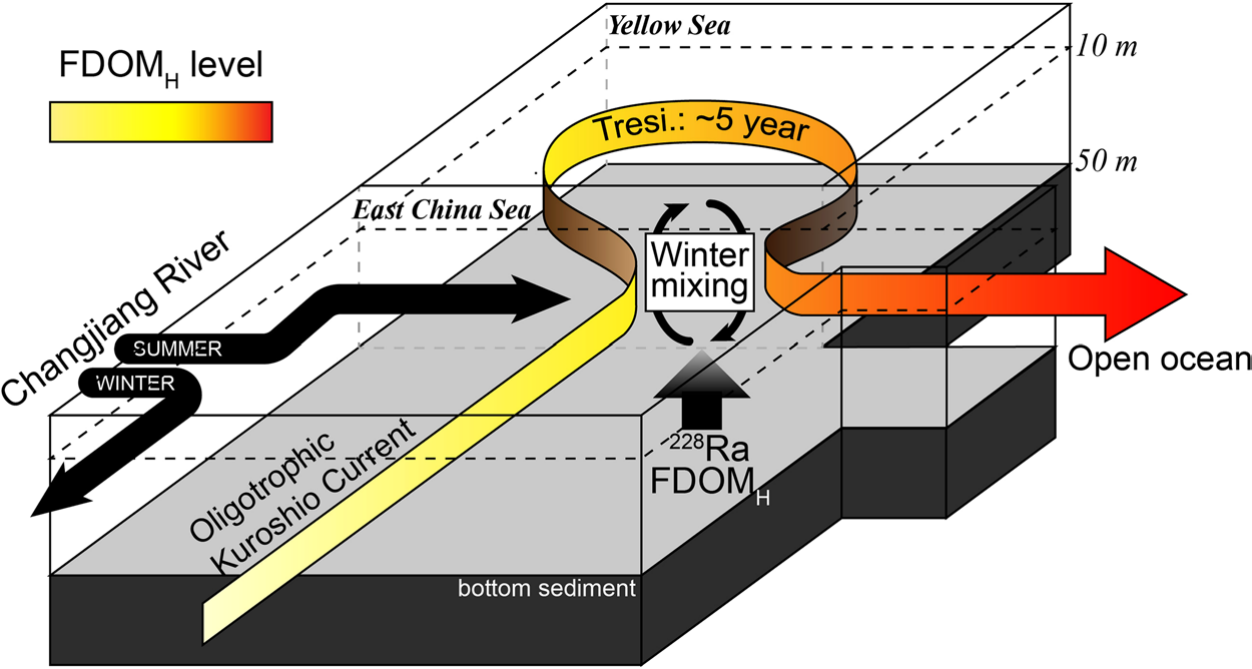
A schematic diagram illustrating the process of the enrichment of FDOM_H_ in the northwestern Pacific continental shelf waters.

Instead, we use ^228^Ra as a tracer of the YSBW because the large enrichment of ^228^Ra by diffusion from bottom sediments has been well documented and it has been utilized as a tracer of water residence times in the Yellow Sea^38,39^. In general, ^228^Ra is very soluble in seawater, and ^228^Ra (half-life: 5.75 years) in the YSBW is produced mainly from particle-reactive ^232^Th (half-life: 1.4 × 10^10^ years) in bottom sediments over the water residence time. The ^228^Ra inputs from rivers and submarine groundwater discharge, relative to the benthic fluxes, are known to be relatively small^39^. Because of this, long-lived ^226^Ra has been used for tracing groundwater inputs as the production of ^226^Ra (half-life: 1622 years) from particle-reactive ^230^Th (half-life: 7.5 × 10^4^ years) in the surface sediments is relatively negligible^38–41^. In this region, higher concentrations of FDOM_H_ were generally observed in pore waters^42^. Thus, we hypothesized that the excess FDOM_H_ and ^228^Ra in the deep layer are continuously produced from bottom sediments and accumulate in the bottom water layer over water residence times of approximately five years^38,40^. The inputs of FDOM_H_ and ^228^Ra produced from bottom sediments through saline groundwater recirculation and those produced from sinking particles are also included in the estimation. The concentrations of FDOM_H_ produced in the continental shelf showed a good positive correlation with ^228^Ra activities soon after the strong typhoon period and the winter (February 2017) (Fig. 4). This trend suggests that the main source of the enriched FDOM_H_ in the YSBW is produced in the continental shelf sediment. The enriched deep FDOM_H_ seems to be well mixed vertically with the surface water because of winter mixing and the typhoon event (Fig. 5). However, no significant correlation was observed between FDOM_H_ and ^228^Ra in the well-stratified surface water layer during the summer of 2012, due to different source inputs between FDOM_H_ and ^228^Ra (Fig. 4).

In our study region, C2 showed a good correlation with salinities across all seasons, without seasonal changes. In general, FDOM_P_ in the river waters known to be derived from terrestrial sources, anthropogenic protein pollution, and local biological production in combination^43^. The Changjiang River is the major source of nutrients and organic substances in this study region throughout the year^44,45^. Thus, the linear trend of FDOM_P_ versus salinity seems to be due to significant biological production in the Changjiang River, relative to that in the continental shelf waters. This trend suggests that photo-bleaching and/or *in situ* production is insignificant during the estuarine mixing processes.

### Estimating the fluxes of FDOM from organic sediments versus rivers

The fluxes of FDOM_H_ produced in the bottom water (*Flux*_*S*_) can be calculated using the following equation:

$$Flu{x}_{s}=\frac{(FDO{M}_{YSBW}-FDO{M}_{SSYW})\times {V}_{YS}}{{T}_{YS}}$$

where *FDOM*_*YSBW*_ and *FDOM*_*SSYW*_ are the endmember values of FDOM (unit: µg QS/L) in the YSBW and the summer surface Yellow Sea water (SSYW), respectively; *V*_*YS*_ is the total water volume (unit: km^3^) of the Yellow Sea; and *T*_*YS*_ is the water residence time of the Yellow Sea (unit: year). The area of the Yellow Sea is 4 × 10^5^ km^2^, and the mean depth is 50 m^38^. The values of *FDOM*_*YSBW*_ were assumed to be 1.8 ± 0.1 QSU for C1 and 0.7 ± 0.1 QSU for C3, the average of high concentrations of FDOM_H_ in the deep Yellow Sea, based on the cross-sectional distributions of FDOM on the continental shelf (Figures S2 and S3). The values of *FDOM*_*SSYW*_ were assumed to be 0.9 QSU for C1 and 0.5 QSU for C3, the estimated concentrations of FDOM in the surface Yellow Sea during the summer at the representative salinity of the Yellow Sea water mass (31.5^39,40^) based on the correlation equation (Figure S4). Then, the fluxes of FDOM_H_ from organic sediments (*Flux*_*S*_) were estimated to be 3.7 (±0.4) × 10^1^ and 1.0 (±0.3) × 10^15^ µg QS/year for C1 and C3, respectively. In this region, the FDOM fluxes from the Changjiang River were estimated to be 8.8 × 10^15^ and 3.8 × 10^15^ µg QS/year for C1 and C3, respectively, by multiplying the extrapolated intercept of the FDOM_H_-salinity relationship and annual river discharge^14^. Therefore, FDOM_H_ production in the continental shelf corresponds to 42% (C1) and 26% (C3) of that from the Changjiang River which is known to be the major FDOM_H_ source in this study region^14^.

In addition to the winter mixing process, typhoons may introduce bottom source FDOM_H_ to the surface water during the summer. After Typhoon, Bolaven, the excess FDOM_H_, relative to that expected from the measured salinity using the FDOM_H_-salinity relationship before the typhoon event, was also observed in the surface water (Fig. 3). Based on the salinity-FDOM_H_ regression equation for the normal period, the average excess concentrations of C1 and C3 during the post-typhoon period were approximately 0.2 ± 0.2 QSU and 0.1 ± 0.1 QSU, respectively. Such FDOM increases following typhoon events are consistent with previous observations in the North Atlantic after Hurricane Gert in September 1999^46^ and the Mid-Atlantic Bight after hurricane-induced vertical mixing^47^. Therefore, either winter or summer mixing by special events can introduce a significant amount of bottom-sourced FDOM into surface waters, which may significantly affect carbon cycles and marine ecosystems.

## Conclusions

The Changjiang River seems to be the major source of FDOM over the northwestern Pacific continental shelf surface waters during the summer. In addition, there is significant input of FDOM from organic sediments resulting in large excess FDOM_H_ during the winter and following typhoon events in surface waters. These bottom source inputs were proven by significant correlations between ^228^Ra and excess FDOM_H_ concentrations. The fluxes of C1 and C3 from organic sediments account for approximately 42% and 26%, respectively, of those originating from the Changjiang River. Our results highlight that the enrichment of FDOM by organic sediment sources can be a key factor controlling the distributions and fluxes of FDOM in the northwestern Pacific continental shelf. Further studies are necessary to investigate the fluxes of FDOM across the continental shelf to the open ocean.

## Materials and Methods

### Sampling

The winter seawater sampling was carried out in the East China Sea from 6 to 17 February 2017 aboard R/V *Onnuri* of the Korean Institute of Ocean Science and Technology (KIOST). The summer seawater sampling for deep FDOM was carried out in the Yellow Sea from 13 to 15 August 2012 aboard *KCG vessel 3011* of the Korea Coast Guard (KCG) (Fig. 1). Seawater samples were collected using 10-L Niskin bottles mounted on a CTD rosette sampler. The hydrographic data were measured using an SBE911-plus CTD profiler (Sea-Bird Electronics Inc., WA, USA). During the sampling cruise in the southern sea off Korea, Typhoon Bolaven, which was classified as Category 4, passed through this study region from 27 to 28 August 2012.

### FDOM analysis

Seawater samples were vacuum-filtered using pre-combusted (450 °C for 4 h) GF/F filters (Whatman, pore size: 0.7 μm). Filtered samples were stored in pre-combusted dark-colored glass bottles. 3D fluorescence spectroscopy was conducted using a fluorescence spectrometer (FS-2, SCINCO, Korea) within 2 weeks after sampling. Excitation-emission matrix spectroscopy (EEMS) analysis was conducted by detecting emission wavelengths (Em) of 250–600 nm at 2-nm intervals, and excitation wavelengths (Ex) of 250–500 nm at 5-nm intervals. The slit widths were set to 10 nm for both excitation and emission monochromators, and the integration time was 50 ms. To prevent any artificial problems with the blank subtraction method, Rayleigh and Raman scatter bands (±15 nm) were eliminated and replaced by the 3D Delaunay interpolation of the remaining adjacent data^48^. The PARAFAC model was applied to identify the peaks of the specific individual fluorophores from 3D EEMS data^49^. PARAFAC analysis for 249 EEMS data was performed using the MATLAB program R2015a (MathWorks, MA, USA) with the DOMFluor toolbox developed by Stedmon and Bro^50^. The three components were statistically validated by split-half analysis (Figure S1). Fluorescence intensities of samples were normalized daily to fluorescence of a quinine sulfate dihydrate (QSU; µg QS/L) diluted in 0.1 N sulfuric acid at a specific wavelength (Ex/Em = 350/450 nm). The variation of its fluorescence was less than 3% of the average value throughout the all measurements. The precision of the measurements was ± 0.01 QSU, and the limit of quantification was 0.14 QSU^9^. The inner filter effect was not corrected because this artifact was negligible for various water samples using the spectrofluorometer^51,52^.

### Ra-228 analysis

The bulk seawater samples (~100 L) were passed through a column of MnO_2_ impregnated acrylic fiber (Mn-fiber) at <1 L min^−1^ to extract Ra isotopes^53^. Mn-fiber samples were hand-rinsed gently to remove any salts and ashed using a muffle furnace at 820 °C for 16 h. The ash was homogenized and transferred to hermetically sealed vials. The samples were then analyzed using a high-purity Germanium well-type detector (CANBERRA Industries Inc., Meriden, CT, USA). The activities for ^228^Ra were determined using the gamma peaks of ^228^Ac (at 911 keV)^54^. Radium activities for all samples were corrected for radioactive decay to the sampling time. Uncertainties of radium isotope activities were computed using an error propagation calculation.

### Data compilation

We also compiled data for ^228^Ra and surface FDOM during the summer season which have been documented from Lee *et al*.^39^ and Kim and Kim^14^, respectively in order to strengthen our arguments. In the case of the FDOM data, the PARAFAC model was re-applied with the compiled raw data set of both seasons.

### Data availability

The datasets analysed during the current study are available from the corresponding author upon reasonable request.

## Electronic supplementary material

Supplementary information
