# Supplementary material for: Significant production of humic fluorescent dissolved organic matter in the continental shelf waters of the northwestern Pacific Ocean
Source: Sci Rep. 2018 Mar 20;8:4887. doi: 10.1038/s41598-018-23299-1 (PMC5861113; doi:10.1038/s41598-018-23299-1)
Supplement: Supplementary file 1 — Supplementary information [file 41598_2018_23299_MOESM1_ESM.docx]

*Scientific Reports*

Supplementary Information for

**Significant production of humic fluorescent dissolved organic matter in the continental shelf waters of the northwestern Pacific Ocean**

Jeonghyun Kim, Hyung-Mi Cho, Guebuem Kim

School of Earth and Environmental Sciences, Seoul National University, 1 Gwanak-ro, Gwanak-gu, Seoul 08826, Korea


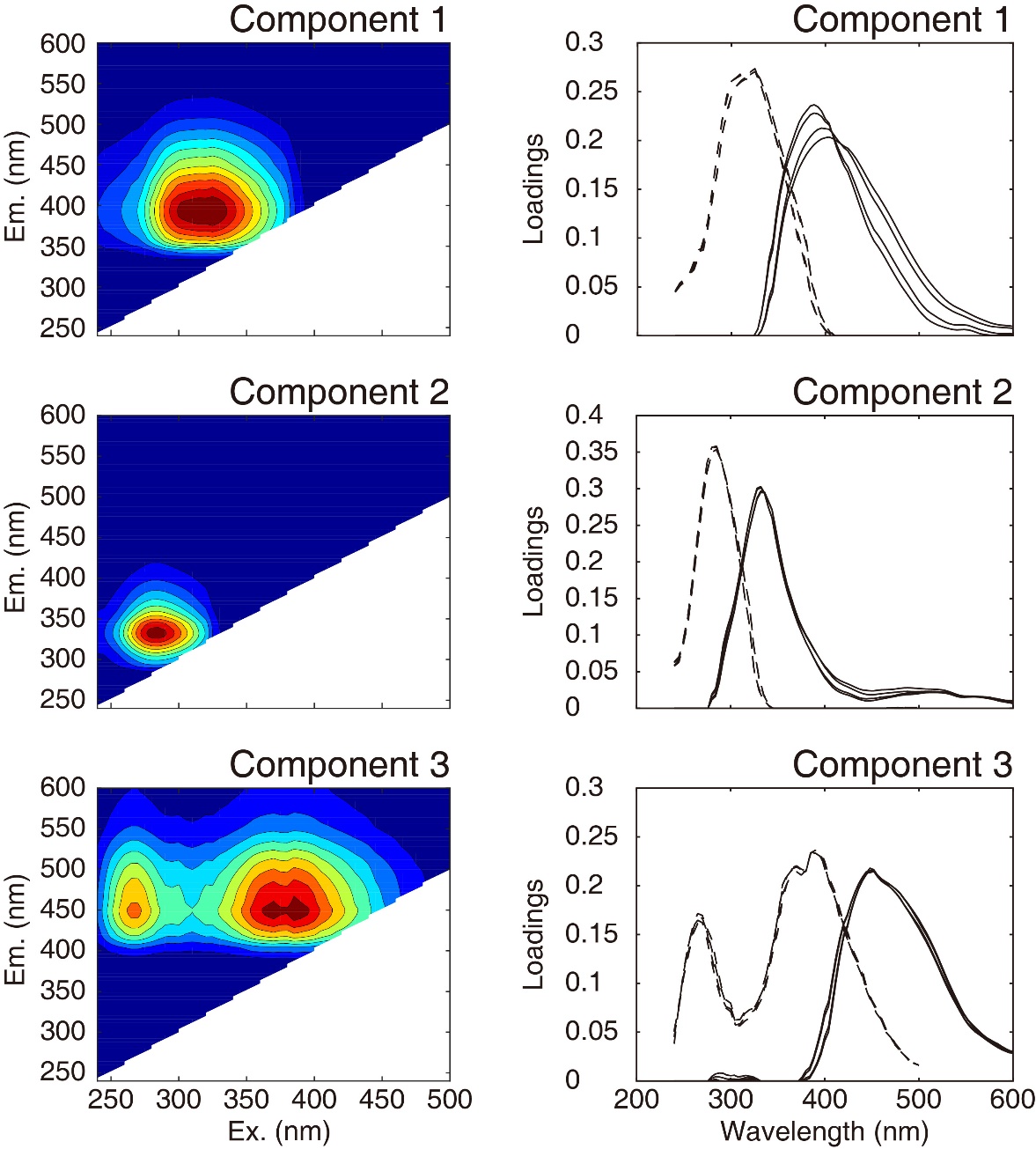


**Figure S1.** Excitation–emission matrix spectroscopy (EEMs) contour plots and loadings of three components (C1, C2, and C3) determined using the Parallel Factor Analysis (PARAFAC) model. The solid line represents the emission wavelength, and the dotted line represents the excitation wavelength.


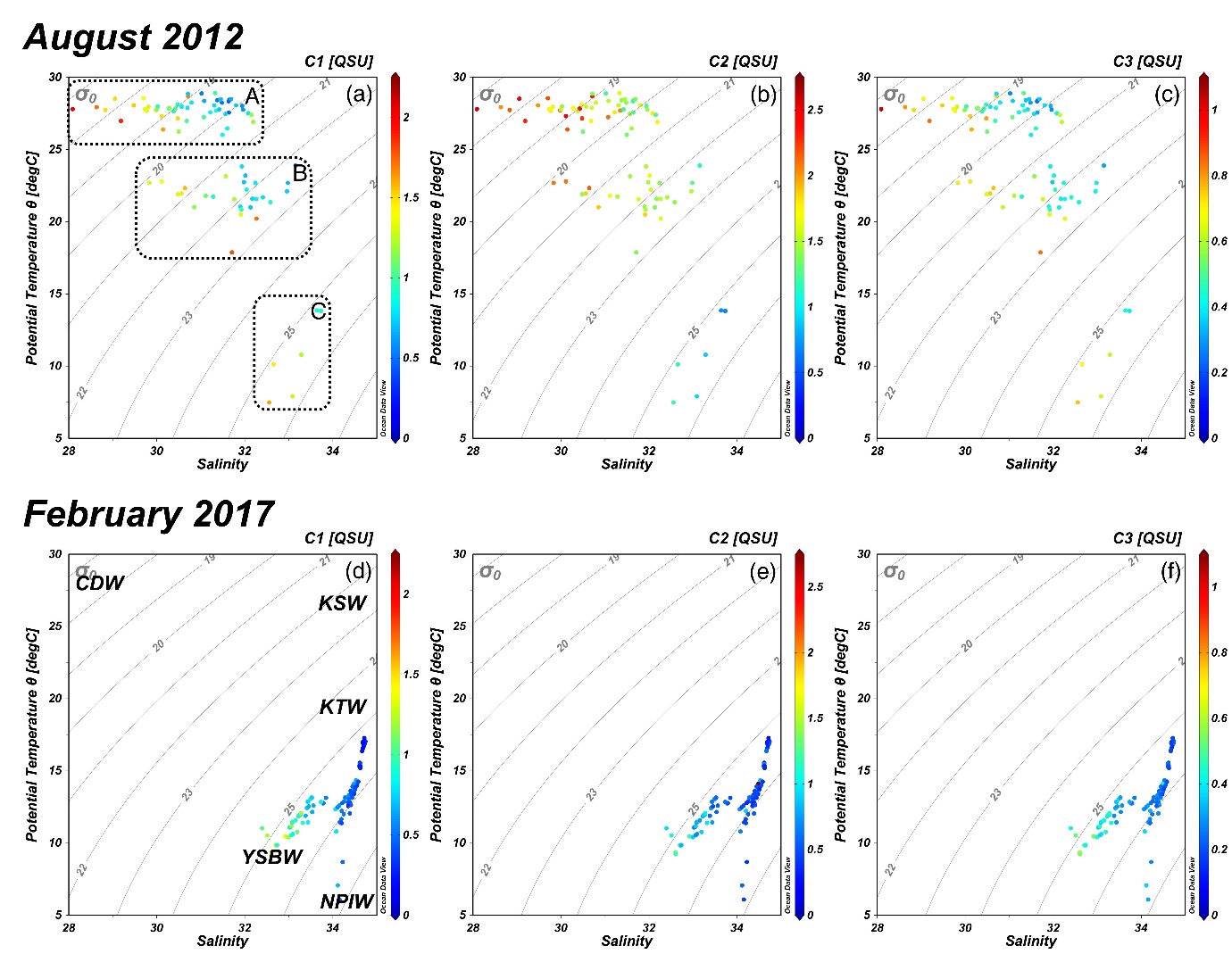


**Figure S2.** Potential temperature versus salinity diagrams (T-S diagram) with the concentrations of the FDOM components (C1, C2, and C3) during August 2012 (a, b, and c) and February 2017 (d, e, and f) (b). Boxes A, B, and C in Figure S1(a) indicate the data for the surface water during summer, the surface water in the post-typhoon periods, and the deep water, respectively. CDW, KSW, KTW, YSBW, and NPIW in Figure S1(b) indicate the Changjiang Diluted Water, Kuroshio Surface Water, Kuroshio Tropical Water, Yellow Sea Bottom Water, and North Pacific Intermediate Water, respectively^1,2^.

**
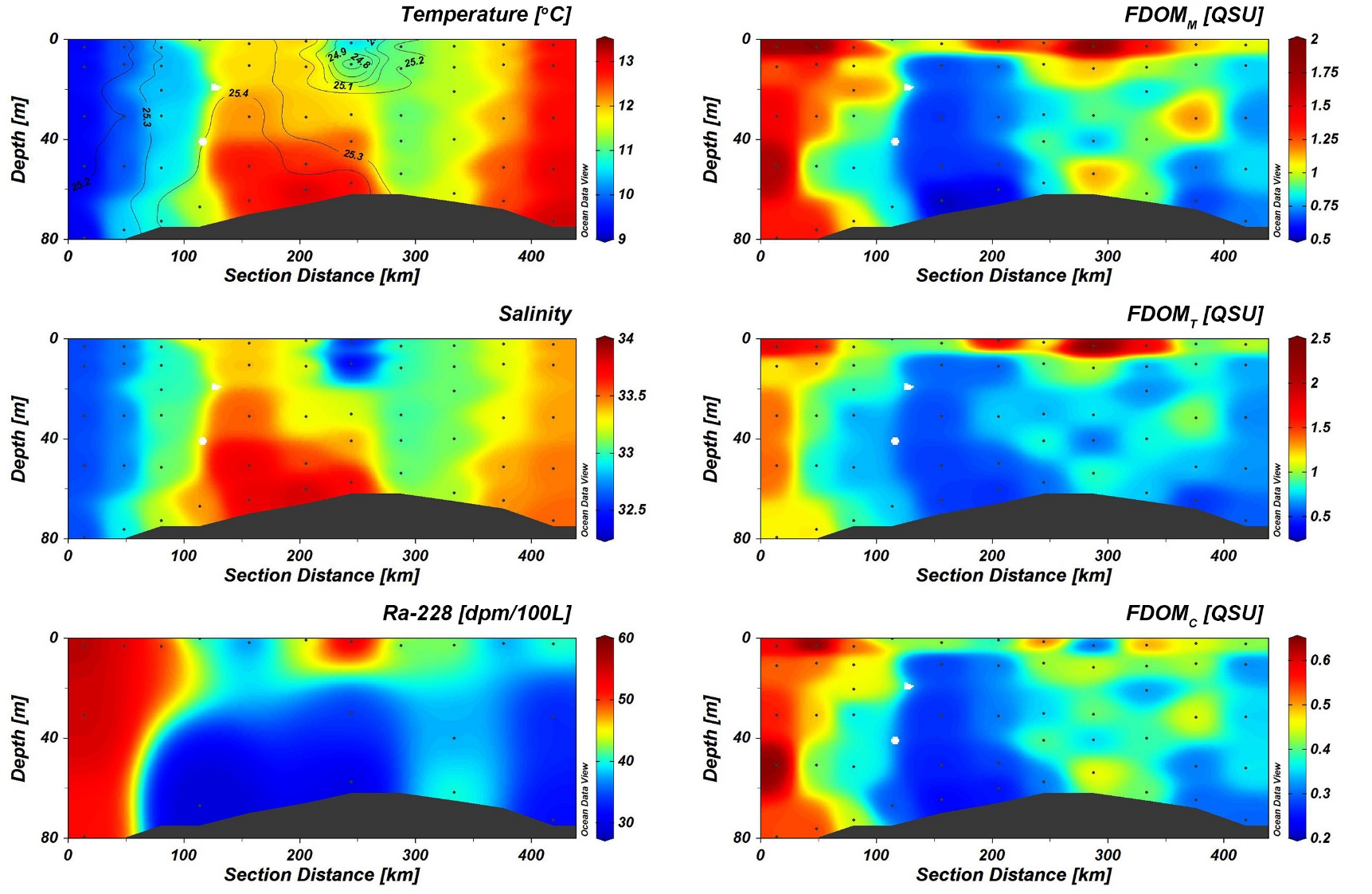
**

**Figure S3.** Cross sections of temperature, salinity, ^228^Ra, and FDOM concentrations across the continental shelf in February 2017. The solid lines on the distribution of temperature represent potential density anomaly (unit: kg/m^3^).

**
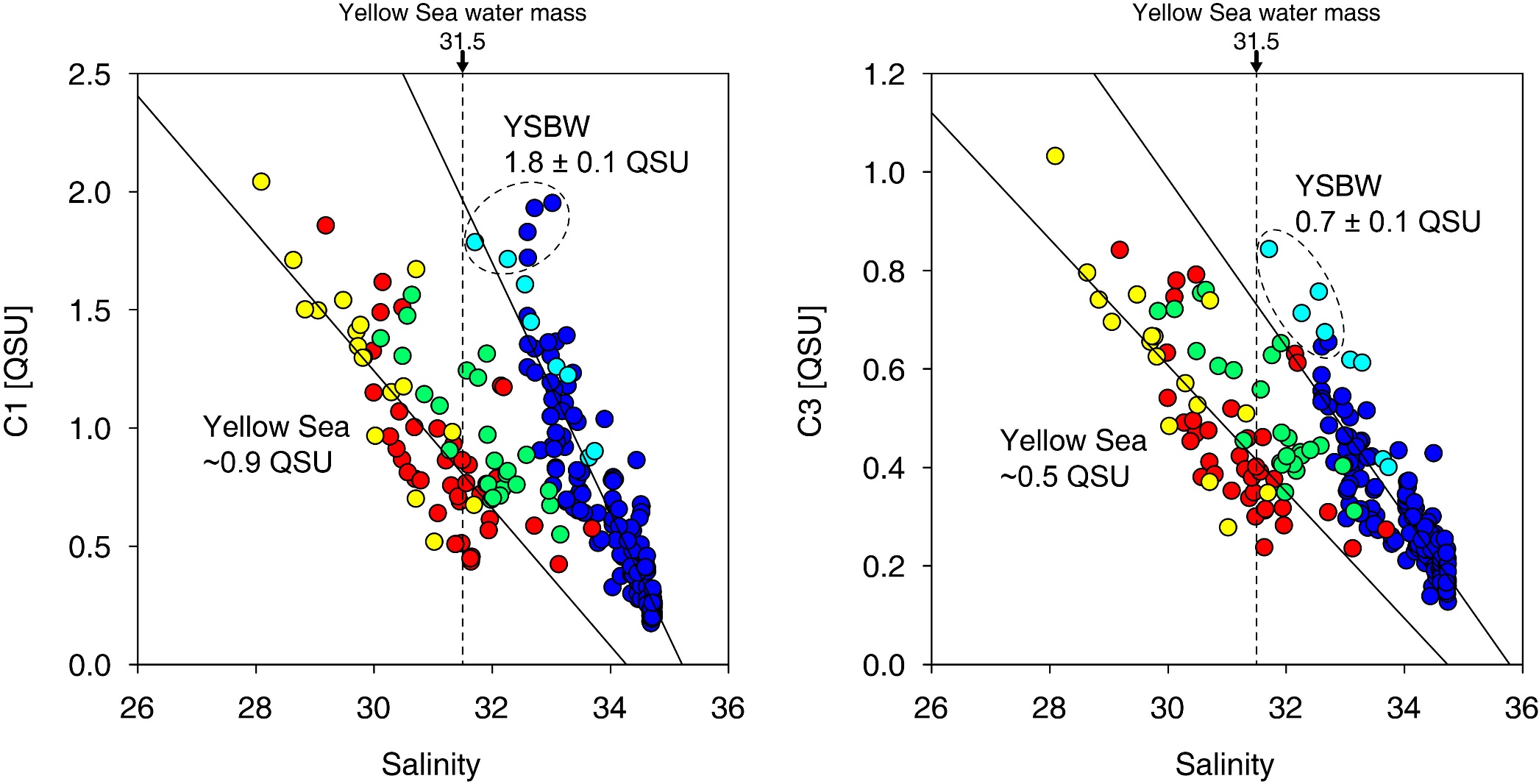
**

**Figure S4.** Schematic diagrams illustrating the estimation of excess FDOM_H_ (C1 and C3) over the Changjiang River source in the northwestern Pacific continental shelf waters. The representative salinity of the Yellow Sea was 31.5 based on Nozaki et al. ^3^ and Lee et al. ^4^. YSBW indicates the Yellow Sea Bottom Water.

**References**

1 Chen, C., Beardsley, R. C., Limeburner, R. & Kim, K. Comparison of winter and summer hydrographic observations in the Yellow and East China Seas and adjacent Kuroshio during 1986. *Continental Shelf Research* **14**, 909-929 (1994).

2 Chen, C. T. A., Ruo, R., Paid, S. C., Liu, C. T. & Wong, G. T. F. Exchange of water masses between the East China Sea and the Kuroshio off northeastern Taiwan. *Continental Shelf Research* **15**, 19-39, doi:<https://doi.org/10.1016/0278-4343(93)E0001-O> (1995).

3 Nozaki, Y., Tsubota, H., Kasemsupaya, V., Yashima, M. & Naoko, I. Residence times of surface water and particle-reactive ^210^Pb and ^210^Po in the East China and Yellow seas. *Geochimica et Cosmochimica Acta* **55**, 1265-1272 (1991).

4 Lee, H., Kim, G., Kim, J., Park, G. & Song, K.-H. Tracing the flow rate and mixing ratio of the Changjiang diluted water in the northwestern Pacific marginal seas using radium isotopes. *Geophysical Research Letters* **41**, 2014GL060230, doi:10.1002/2014GL060230 (2014).
